# High-Throughput Phenotyping of Wheat and Barley Plants Grown in Single or Few Rows in Small Plots Using Active and Passive Spectral Proximal Sensing

**DOI:** 10.3390/s16111860

**Published:** 2016-11-05

**Authors:** Gero Barmeier, Urs Schmidhalter

**Affiliations:** Chair of Plant Nutrition, Department of Plant Sciences, Technical University of Munich, Emil-Ramann-Str. 2, Freising 85354, Germany; barmeier@wzw.tum.de

**Keywords:** border-row effect, high-throughput, phenomics, phenotyping, plant breeding, plot design, precision, spectral proximal sensing

## Abstract

In the early stages of plant breeding, breeders evaluate a large number of varieties. Due to limited availability of seeds and space, plot sizes may range from one to four rows. Spectral proximal sensors can be used in place of labour-intensive methods to estimate specific plant traits. The aim of this study was to test the performance of active and passive sensing to assess single and multiple rows in a breeding nursery. A field trial with single cultivars of winter barley and winter wheat with four plot designs (single-row, wide double-row, three rows, and four rows) was conducted. A GreenSeeker RT100 and a passive bi-directional spectrometer were used to assess biomass fresh and dry weight, as well as aboveground nitrogen content and uptake. Generally, spectral passive sensing and active sensing performed comparably in both crops. Spectral passive sensing was enhanced by the availability of optimized ratio vegetation indices, as well as by an optimized field of view and by reduced distance dependence. Further improvements of both sensors in detecting the performance of plants in single rows can likely be obtained by optimization of sensor positioning or orientation. The results suggest that even in early selection cycles, enhanced high-throughput phenotyping might be able to assess plant performance within plots comprising single or multiple rows. This method has significant potential for advanced breeding.

## 1. Introduction

In early selection cycles in plant breeding, large numbers of plants need to be tested, and in agronomic field testing, extensive evaluation of plant performance is also required. Both seed availability and financial constraints frequently necessitate testing of plants in one or several rows, with space limitations also contributing to a need for small plot sizes. Limited resources, therefore, necessitate smaller plots [1]. In general, plot size depends on the type of experiment, breeding objectives, available resources and equipment, and the stage of breeding [2]. However, plot sizes vary substantially among field trials, ranging from single-plant plots to plots of several hundred square meters [3]. Small plots with 2–3 rows are usually used in early stages of breeding projects to evaluate varieties quickly and inexpensively. In advanced selection cycles, when selection for yield also occurs, larger plots are used, and the data may be collected from middle rows [2]. Such plot trials, thus, aim to predict the performance of the tested varieties by mimicking agricultural field conditions. However, such predictions may be inaccurate since the phenotypic performance of plants grown at different spacing may differ from that of plants grown using conventional agricultural practices [1]. The small size of plots may be disadvantageous because border row effects are known to influence yield. Depending on the type of plot trial, external rows may show increased yield [4] due to increased tillering [5].

Since the management of field trials comprising a large number of plots is highly labour-intensive, new methods, such as spectral proximal sensing for the estimation of specific plant traits, are becoming increasingly more important [6,7]. However, commercially available spectral proximal sensors, such as the GreenSeeker (NTech Industries Inc., Ukiah, CA, USA), as well as hyperspectral passive sensors [8,9], were originally designed and tested for field conditions and not specifically for small-plot testing. Therefore, assessment of the sensors in plot trials is of great importance, and particular attention should be paid to the evaluation of the sensed areas. Such evaluation requires the consideration of technical aspects, such as sensor-target distances, and the influences of environmental conditions, such as light intensity and temperature [10,11]. Sensors should be compatible with various plot designs, which ultimately requires a match between the sensors’ field of view and the tested target.

**Figure 1:**
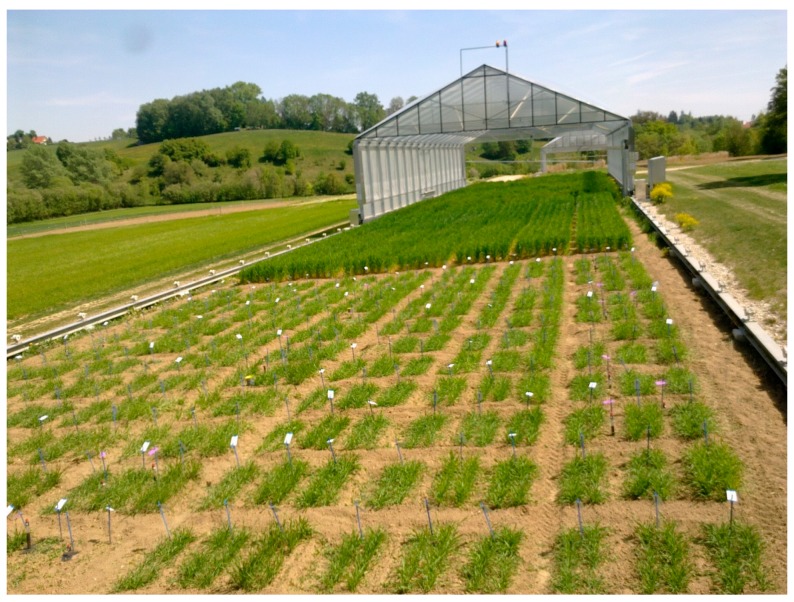
Field trials within a rain-out shelter platform, illustrating different row designs for spring barley grown in two rows (**foreground**) and for winter barley grown in six rows (**background**).

Field trials comprising different plot designs (Figure 1) and cropped with one or multiple species, are challenging to evaluate, and their potential for assessment by proximal sensing needs to be determined.

Non-invasive assessments of small plots must take into account uneven growth due to differences in the light availability or enhanced nutrient and water uptake. It is also important to consider whether middle rows are to be assessed preferentially or an integral assessment of the whole plot is desired. Additionally, reflectance sensors differ in their spectral fields of view, ranging from linear to oval and circular shapes [8,11], and are also influenced whether the sensor’s orientation is parallel or opposed to the row.

Numerous studies have described border row effects [12] and the advantages and disadvantages of different field trial designs [4,13,14,15], in addition to comparing different spectral sensors [8,11,16]. To the best of our knowledge, no studies have compared the performances of active and passive sensors in assessing the single or multiple rows used in breeding or agronomic experiments; such a comparison was the goal of this work. Since wide plots with many rows have already been tested repeatedly [7,10,16,17], the purpose of this work is to assess the performance of active and passive spectral sensing in plot designs of one, two, three, and four rows, like those commonly used in breeding trials for wheat and barley. Previous studies have shown no difference in spectral performance when assessing plots with six or more rows. In this work, the influence of different plot designs on biomass and grain yield is illustrated, highlighting the performance of spectral sensors in non-invasive detection of these traits.

## 2. Materials and Methods

### 2.1. Plot Experiments and Biomass Sampling

Field experiments were conducted at the Dürnast Research Station of the Technical University of Munich (TUM) in Germany (11°41′60″ E, 48°23′60″ N) in 2014. The soil is a mostly homogeneous Cambisol of silty clay loam texture, the annual precipitation is approximately 800 mm, and the average temperature is 7.5 °C.

**Figure 2:**
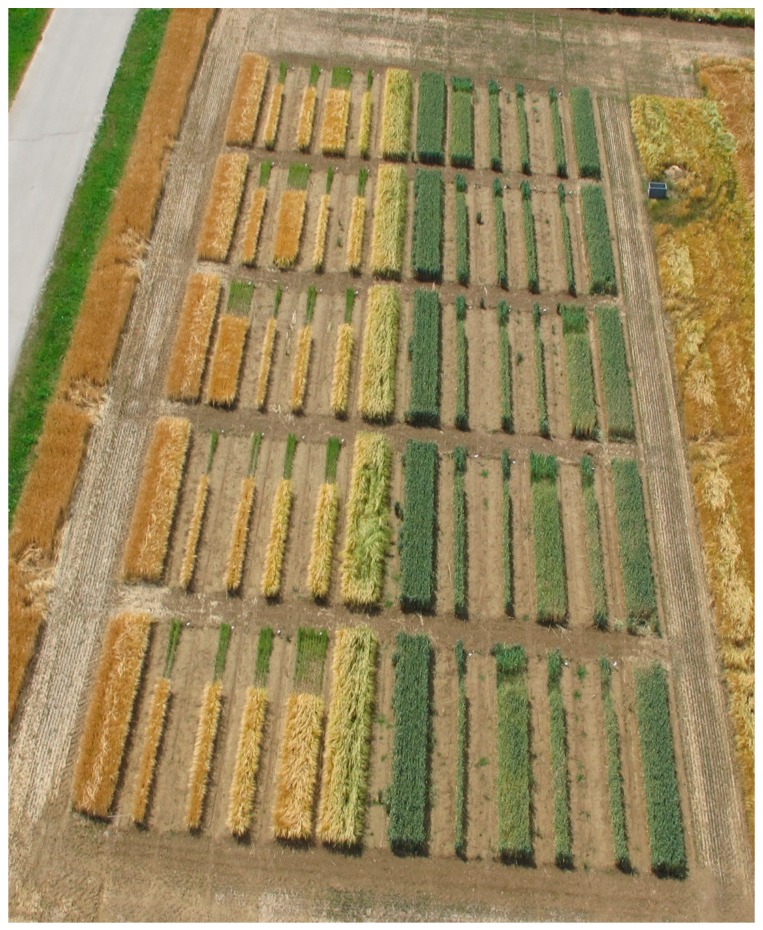
UAV image of the field trial. Different plot designs, including one-, two-, three- and four-row designs, were tested using winter wheat and winter barley as crops.

A randomized block design was used to test both barley (*Hordeum vulgare* L. cv. Sandra) and wheat (*Triticum aestivum* L. cv. Kerubino), with four planting-row designs and four replicates, totalling 40 plots (Figure 2).

The plots were 10 m in length. The planting-row designs consisted of plots with a single row, plots with two rows with 25-cm row spacing, and plots with three and four rows with 12.5-cm row spacing.

The wider 25-cm row spacing is frequently used for testing the performance of barley, whereas the narrower spacing of 12.5 cm is commonly used for testing wheat in breeding nurseries in Germany.

Fungicide treatments followed local recommendations. Weeds were removed by hand to remove possible bias in interpreting the results. Nitrogen fertilizer was applied in a single dose at ZS 15 [18] as ammonium sulphate using the nitrification inhibitor ENTEC [19] with 150 kg·N/ha and 60 kg·S/ha in amounts corresponding to the different numbers of rows.

Biomass samplings were performed at Zadoks stage 32 [18] (stem elongation), ZS 60 (anthesis) and ZS 85 (soft dough) by cutting plants above the ground along 1 m of each row. The fresh biomass was immediately determined in the field by weighing, and a subsample was oven-dried at 60 °C for three days until a constant dry weight was reached. The nitrogen content was determined by mass spectrometry using an isotope ratio mass spectrometer with an ANCA SL 20-20 preparation unit (Europe Scientific, Crewe, UK) and nitrogen uptake was calculated by multiplying plant dry weight by N concentration.

### 2.2. Spectral Reflectance Measurements

**Figure 3:**
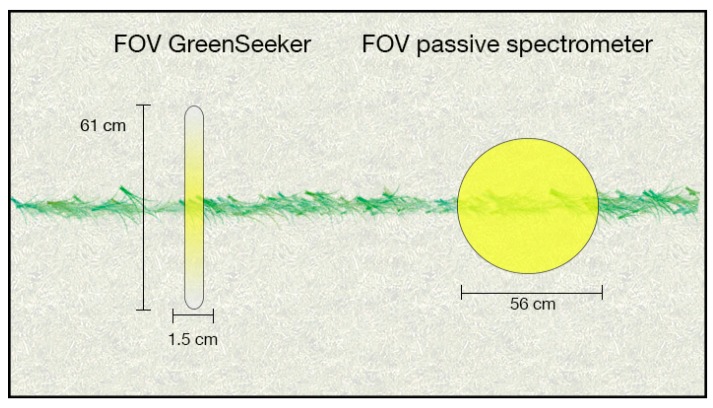
Illustration of different shapes of the sensors’ fields of view (FOV) in single-row trials. Yellow colour indicates decreasing light intensity in the periphery of the LED-based GreenSeeker (unpublished data).

**Figure 4:**
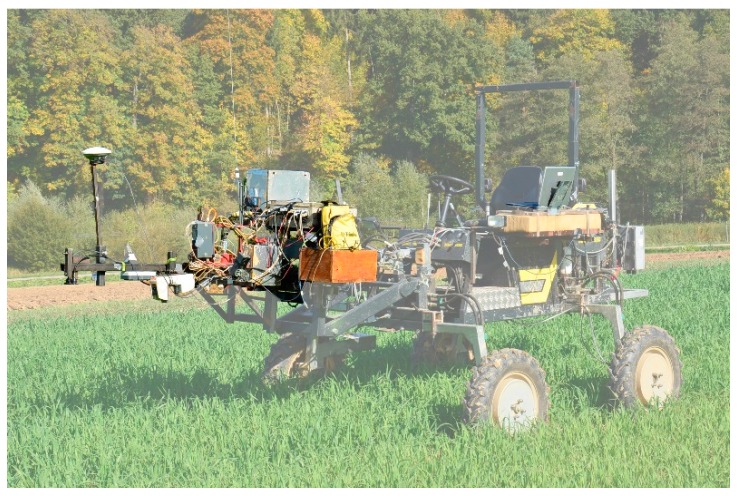
Sensor carrier PhenoTrac IV, customized by the Chair of Plant Nutrition from the Technical University of Munich.

Two different sensors were used in this study: a passive bi-directional reflectance sensor customized by the Chair of Plant Nutrition from the TUM and a GreenSeeker RT100 (NTech Industries Inc., Ukiah, CA, USA). The passive bidirectional reflectance sensor contained two Zeiss MMS1 silicon diode array spectrometers with a spectral detection range from 300 to 1000 nm and a bandwidth of 3.3 nm [20]. One spectrometer was linked to a diffuser detecting the solar radiation as a reference signal. The second spectrometer measured the canopy reflectance with a field of view (FOV) of 12° within a circular shape, resulting in a sensor-target distance of approximately 1 m with a FOV of approximately 0.28 m². The GreenSeeker used two LEDs as a light source and detected the reflection of visible (656 nm, ~25 nm band width) and near-infrared (774 nm, ~25 nm band width) spectral regions. The FOV of the GreenSeeker was a strip of approximately 61 by 1.5 cm, resulting in a scanned area of approximately 0.009 m² (Figure 3) [8]. Both sensors were mounted on a frame in front of the PhenoTrac IV [9], a sensor-vehicle platform customized by TUM (Figure 4), at a height of approximately 1 m above the plant canopy. The measurements were conducted under clear sky conditions at noon, one hour after biomass sampling. Afterwards, the normalized difference vegetation index (NDVI) [21] was calculated as follows:

$$NDVI=\frac{R774\;\mathrm{nm}-R656\;\mathrm{nm}}{R774\;\mathrm{nm}+R656\;\mathrm{nm}}$$

In addition, the simple ratio (SR) was determined as follows:

$$SR=\frac{R774\;\mathrm{nm}}{R656\;\mathrm{nm}}$$

and three simple ratios were further selected based on a contour map analysis depicting all dual wavelength combinations:

$$SR=\frac{R800\;\mathrm{nm}}{R770\;\mathrm{nm}}$$

$$SR=\frac{R820\;\mathrm{nm}}{R755\;\mathrm{nm}}$$

$$SR=\frac{R720\;\mathrm{nm}}{R400\;\mathrm{nm}}$$

### 2.3. Statistical Analysis

R version 3.1.2 (R Foundation for Statistical Computing, Vienna, Austria) was used for calculating the coefficients of variation, the standard errors, and linear regressions between the data obtained from the sensors and the destructive measurements. An analysis of variance (ANOVA) with Tukey’s HSD (honest significant difference) test (*p* ≤ 0.05) was used to group and differentiate between planting-row designs. For an enhanced analysis of optimized wavelength combinations, a contour map analysis was used.

## 3. Results

### 3.1. Effects of Different Row Designs on Plant Fresh and Dry Weight, Aboveground Biomass Nitrogen Uptake, and Grain Yield

Even at early stages of development, the different row designs exhibited clear differences. Compared to a reference plot with 10 rows, the single-row plots showed an increase in fresh weight of 124% for barley and 90% for wheat at ZS 32. At ZS 65 and ZS 85, this difference grew to an increase of 235% in wheat biomass in the single-row plot design compared to the 10-row plot. Mean values of the destructively-assessed plant parameters are given in Table 1. Significant differences (*p* ≤ 0.05) were found between the designs in aboveground fresh and dry weights, as well as in the calculated N uptake; however, no differences in aboveground plant N content were found. Especially for wheat at early stages of growth, no distinction among the one-, two-, and three-row designs, or among the two-, three-, and four-row designs was found for plant fresh weight or dry weight. These designs were characterized by excessive tillering. This trend remained until ZS 85, when the two-, three-, and four-row designs still had comparable biomasses. Even the one-row design showed increases of up to 75% in plant dry weight compared to the two-row design.

Barley showed similar responses, though the four-row design differed significantly from the two-row design at ZS 32 and 65. At ZS 85, however, the two-, three-, and four-row designs all had similar plant fresh weight and dry weight.

A statistical grouping of grain yields showed a high compensatory performance, particularly for wheat. No difference was observed among the two-, three-, and four-row designs. Compared to the ten-row plots, grain yields gradually increased with decreasing number of rows.

### 3.2. Relationship between Plant Parameters Obtained from Combination of the Four Plot Designs and Spectral Reflectance Measurements

Relationships between sensor measurements and four plant parameters for the combined row designs at three sampling dates are given in Table 2. In general, the passive spectrometer showed closer linear relationships between selected spectral reflectance indices and plant parameters of both species than did the active sensor. The GreenSeeker showed a closer relationship only for wheat at anthesis. Since neither sensor could detect all biomass parameters from wheat at ZS 85 with the vegetation indices available from the GreenSeeker, a contour map method that allowed testing of all possible dual reflectance indices from the passive spectrometer was further evaluated to find whether enhanced vegetation indices could be obtained. These indices, R820/R755 for barley and R720/R400 for wheat, resulted in markedly improved relationships in later growth stages.

Compared to the simple ratio R774/R656, the NDVI showed reduced coefficients of determination caused by saturation effects. In this regard, the simple ratios were less sensitive. For the N content, only weak relationships were obtained.

**Figure 5:**
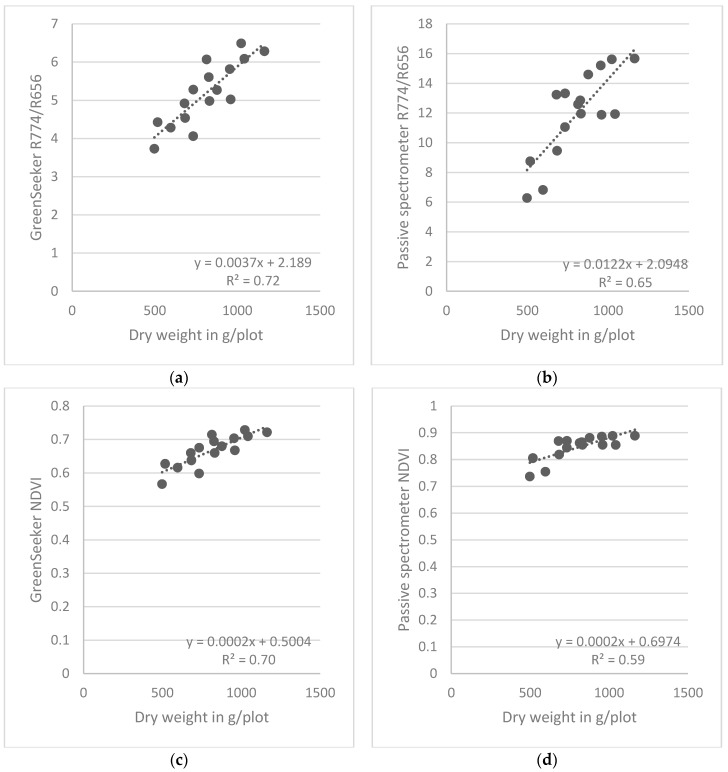
Relationships between spectral indices derived from the two types of sensors and plant dry weight at ZS 65 for wheat, obtained from linear regressions combining the four different row designs.

**Figure 6:**
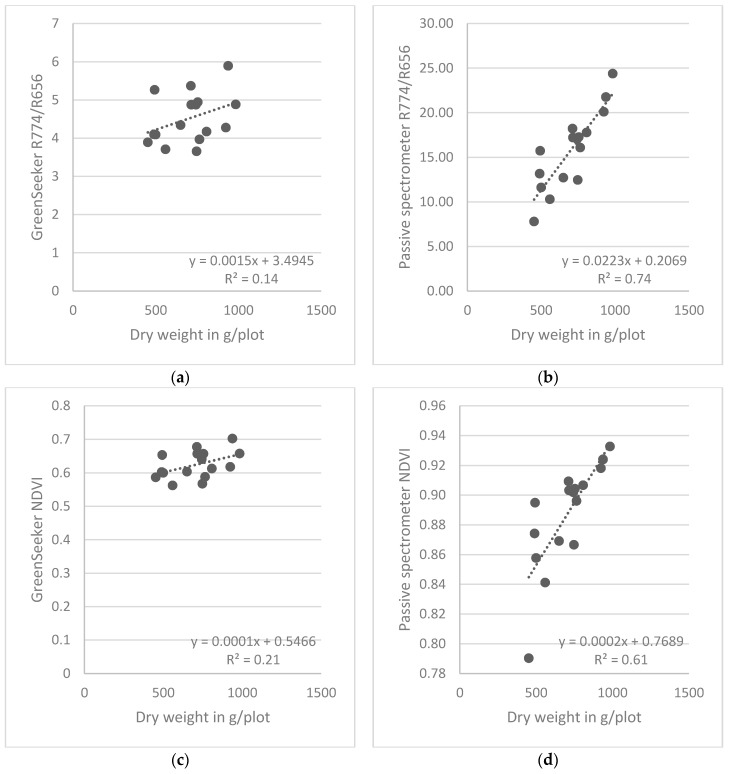
Relationships between spectral indices derived from the two types of sensors and plant dry weight at ZS 65 for barley, obtained from linear regressions combining the four different row designs.

Figure 5 and Figure 6 depict results from linear regressions for the combined row designs for barley and wheat, respectively. The spread of the regression points for the three sampling dates made it necessary to consider each sampling date separately. For barley, the results from the GreenSeeker demonstrated considerable scatter, and the regressions of both the NDVI and the simple ratio were considerably less similar than were the same indices obtained for wheat.

## 4. Discussion

Border row effects, which cause enhanced growth of plants in border rows, have long been well-known, and recommendations for their avoidance, such as harvesting border rows and front sides separately [3], have been reported. However, due to the small numbers of seeds and limited resources available in early selection cycles, plots sharing one to three rows are common and yield estimates are, therefore, biased [2,4]. This is of lesser importance in early selection cycles that focus on the overall performance of varieties, but it should be avoided in later cycles due to competitive effects of neighbouring plants. Still [12] mentioned that the results of small plots are not representative and that there is a need for multi-row plots to simulate field conditions. This agrees with the findings of our study. At all three growth stages, both wheat and barley showed relatively higher fresh and dry weights, as well as greater nitrogen uptake, in single and multiple-row plots than in a plot comprising 10 rows. However, these results cannot be generalized since only single varieties of each species were tested. Further research of the performance of multiple cultivars in small plots needs to be done.

Several authors [6,17,22] have demonstrated that spectral proximal sensing is a suitable tool for breeders and plant scientists to evaluate plant parameters in a non-destructive and high-throughput manner. Studies performed with wide plots of 10 rows demonstrated comparable or, frequently, superior performance of passive sensors compared to active sensors, including the one tested in this study, for wheat [8], maize [10], and barley [16]. These sensors were also tested in different environments by considering the effects of temperatures, light intensity, and surface conditions [11,23]. However, no previous study had tested the performance of spectral proximal sensors in different plot designs. The results showed decreasing spectral reflectance in the one- and two-row plots, indicating an interfering signal received by the sensor. This was most likely due to the higher fraction of soil in the sensor’s FOV. Chemical analysis of the harvested plant material and visual scoring of the plots indicated that neither nutrient deficiencies nor plant diseases occurred in the different plots, and weeds and other objects were manually removed before each measurement. Therefore, it can be concluded that spectral information from bare soil interfered with the spectral sensing of plants, particularly at early growth stages. In later growth stages, distances between sensors and soil increase resulting in a reduced influence of the soil. The GreenSeeker, in particular, with its extended FOV of 1.5 × 61 cm (Figure 3), may be more susceptible to spectral information from the soil in the one- or two-row plots, in which the planted rows were 15 and 35 cm apart, respectively, especially at early stages of growth.

The one-row design covered approximately 25% of the measurement field of the GreenSeeker, whereas this value was approximately 34% for the passive spectrometer. For the two-row design, these values were approximately 57% for the GreenSeeker and 80% for the passive spectrometer. In addition, the light intensity decreases on the periphery of the GreenSeeker, which leads to lower reflection values. Kim et al. [23] showed that the best performance was obtained in central positions within 30 cm of the light strip. Previous research has indicated that the intensity of LED light emitted decreases with increasing distance [10,11]. As a result, the crop stand is not entirely perceived. This is in contrast to passive sensing, which uses the sun as a light source, the intensity of which does not appreciably decrease within the crop stand. However, this may increase the likelihood that passive sensing will detect information from the soil surface in less dense crop stands.

The results from this study also show that fresh and dry weights do not increase linearly in plots with different numbers of rows, with the largest values observed in the one-row design. It is likely that optimized light conditions, together with improved nutrient and water supply, enhanced growth in border rows or in designs with fewer rows. Since only one cultivar of each species was investigated, different performances of other cultivars cannot be excluded.

A comparison of the performance of the sensors and evaluation of the best performing indices revealed that the best results were obtained from the passive sensor with the indices R774/R656 and R800/R770. In agreement with previous results [8], saturation effects became apparent for the index NDVI independent of the sensor. The passive hyperspectral sensor generally outperformed the active sensor, with superior performance of the active sensor found only for wheat at anthesis. Active sensors have the advantage of being independent of light conditions, enabling their use at night, though the bi-directional passive sensor used in this study does allow compensation for changes in light conditions in the day.

Overall, the results show that spectral sensing can be carried out quite successfully in plot designs with few rows; however, some further optimization is still needed, particularly for single rows. The sensors’ FOV did not optimally match such a design, offering one avenue for improvement. For example, the GreenSeeker could be aligned along single rows, while the passive sensor could be positioned closer to the plants, thus covering a higher fraction of the plants’ area because the measurements are not distance dependent. Still, superior performance of the passive sensor has been demonstrated for plot designs with two, three, or four rows.

Taken together, these results suggest that enhanced high-throughput spectral sensing can be used in plot designs with few rows, thereby allowing the evaluation of the performance of varieties or cultivars in early selection cycles. Since early selection cycles, in particular, evaluate many hundreds or thousands of varieties, a highly interesting potential for enhanced breeding is indicated. However, neighbouring effects due to different varieties’ being in close contact with each other should be considered or avoided. Follow-up work should address the feasibility to extend these findings to an extended set of cultivars or varieties representing different species.

## Figures and Tables

**Table 1:** Destructively-assessed values of aboveground plant fresh and dry weight, N content, and aboveground nitrogen uptake of wheat and barley plants as obtained from different plot designs. The plot designs included 1, 2, 3, 4, or 10 rows, and samples were collected from plants at three different stages of development (ZS 32, 65, and 85). Coefficients of variation, standard errors of the means, and plant parameters per plot for the different row designs are indicated, with each value representing the average of four replicates. Rankings are derived from Tukey’s HSD-Test, are indicated at *p* ≤ 0.05 indicating differences within rows. Different letters (a,b,c,d) denote significant differences.

Variant			One-Row				Two-Row			
			Fresh-Weight (g)	Dry-Weight (g)	N-Content (%)	N-Uptake (g)	Fresh-Weight (g)	Dry-Weight (g)	N-Content (%)	N-Uptake (g)
Barley	ZS 32	Means	1113 ^a^	243.6 ^a^	2.9 ^a^	7.1 ^a^	1607.1 ^ab^	372.4 ^ab^	2.5 ^a^	9.4 ^ab^
		CV	0.14	0.20	0.06	0.21	0.12	0.12	0.08	0.13
		SE	78.58	23.82	0.09	0.75	95.15	21.97	0.11	0.62
	ZS 65	Means	1626.2 ^a^	483.3 ^a^	1.7 ^a^	8.2 ^a^	2180.7 ^ab^	666.9 ^ab^	1.7 ^a^	11.9 ^a^
		CV	0.14	0.04	0.09	0.10	0.13	0.11	0.12	0.22
		SE	117.60	9.31	0.08	0.43	144.01	35.75	0.11	1.29
	ZS 85	Means	1745.9 ^a^	706.7 ^a^	1.2 ^a^	8.8 ^a^	2295.5 ^b^	936.9 ^ab^	1.1 ^a^	10.8 ^a^
		CV	0.25	0.22	0.06	0.22	0.19	0.15	0.13	0.25
		SE	218.73	78.50	0.04	0.97	221.52	69.72	0.08	1.35
Wheat	ZS 32	Means	463.2 ^a^	109.6 ^a^	2.6 ^a^	2.8 ^a^	842 ^ab^	190.9 ^ab^	2.5 ^a^	4.7 ^ab^
		CV	0.10	0.08	0.04	0.09	0.09	0.06	0.05	0.09
		SE	23.50	4.40	0.05	0.13	36.44	5.60	0.06	0.22
	ZS 65	Means	1820 ^a^	574.1 ^a^	1.8 ^a^	10.5 ^a^	2584 ^ab^	767.9 ^ab^	1.6 ^a^	12.5 ^a^
		CV	0.06	0.13	0.04	0.17	0.10	0.08	0.08	0.12
		SE	58.80	36.74	0.03	0.88	129.08	32.13	0.07	0.75
	ZS 85	Means	2111.7 ^a^	971.2 ^a^	0.8 ^a^	8.4	2994.1 ^ab^	1383.9 ^b^	0.9 ^ab^	13
		CV	0.02	0.06	0.06	0.06	0.04	0.04	0.17	0.17
		SE	21.54	28.85	0.03	0.26	61.88	24.95	0.08	1.14
**Variant**			**Three-Row**				**Four-Row**			
			**Fresh-Weight (g)**	**Dry-Weight (g)**	**N-Content (%)**	**N-Uptake (g)**	**Fresh-Weight (g)**	**Dry-Weight (g)**	**N-Content (%)**	**N-Uptake (g)**
Barley	ZS 32	Means	1959.2 ^ab^	425.4 ^bc^	2.9 ^a^	12.5 ^ab^	2396.7^ b^	545.7 ^c^	2.7 ^a^	14.7 ^b^
		CV	0.07	0.08	0.09	0.17	0.16	0.14	0.11	0.16
		SE	66.06	17.83	0.13	1.05	189.86	38.82	0.15	1.16
	ZS 65	Means	2595.5 ^b^	799.8 ^b^	1.8 ^a^	15 ^a^	2882.7 ^b^	857.3 ^b^	1.8 ^a^	16.2 ^a^
		CV	0.11	0.10	0.08	0.19	0.17	0.12	0.23	0.33
		SE	149.18	40.01	0.08	1.43	239.40	51.92	0.22	2.65
	ZS 85	Means	2663.9 ^b^	1059.5 ^ab^	1.3 ^a^	14.3 ^a^	2984.6 ^b^	1143.7 ^b^	1.2 ^a^	14.6 ^a^
		CV	0.21	0.17	0.12	0.29	0.12	0.07	0.15	0.21
		SE	278.86	91.74	0.08	2.11	177.28	41.60	0.09	1.56
Wheat	ZS 32	Means	949.9 ^bc^	200.9 ^ab^	2.5 ^a^	5.1 ^bc^	1163.2 ^c^	266.3 ^c^	2.6 ^a^	7 ^c^
		CV	0.17	0.12	0.01	0.12	0.12	0.09	0.08	0.16
		SE	79.89	11.88	0.01	0.30	72.34	12.46	0.11	0.57
	ZS 65	Means	2931.7 ^bc^	887.2 ^b^	1.8 ^a^	16.4 ^a^	3406.7 ^c^	1004.4 ^b^	1.8 ^a^	18.5 ^ab^
		CV	0.14	0.14	0.10	0.23	0.08	0.10	0.13	0.22
		SE	207.99	60.37	0.09	1.87	139.37	52.70	0.12	2.07
	ZS 85	Means	2822.7 ^b^	1338.3 ^b^	0.8 ^ab^	10.6	3347.8 ^b^	1562.5 ^b^	0.9 ^ab^	14.2
		CV	0.17	0.18	0.08	0.13	0.12	0.11	0.14	0.21
		SE	243.30	121.83	0.03	0.71	195.63	84.89	0.06	1.51
**Variant**			**Complete Plot (10 Row)**			
			**Fresh-Weight (g)**	**Dry-Weight (g)**	**N-Content (%)**	**N-Uptake (g)**
Barley	ZS 32	Means	4631.8 ^c^	1085.5 ^d^	2.4 ^a^	26.8 ^c^
		CV	0.09	0.09	0.11	0.14
		SE	198.34	50.32	0.14	1.92
	ZS 65	Means	5254.5 ^c^	1809.9 ^c^	1.8 ^a^	34 ^b^
		CV	0.13	0.16	0.14	0.31
		SE	352.90	145.62	0.13	5.27
	ZS 85	Means	5843.7 ^c^	2403.8 ^d^	1.3 ^a^	31.4 ^b^
		CV	0.10	0.12	0.09	0.13
		SE	297.01	139.30	0.06	2.03
Wheat	ZS 32	Means	2773.2 ^d^	574.7 ^d^	2.7 ^a^	15.3 ^d^
		CV	0.17	0.15	0.08	0.08
		SE	231.69	42.96	0.11	0.58
	ZS 65	Means	6420.5 ^d^	1760.2 ^c^	1.7 ^a^	30.5 ^b^
		CV	0.08	0.09	0.09	0.14
		SE	255.45	75.08	0.08	2.11
	ZS 85	Means	6000 ^c^	2896 ^c^	0.6 ^b^	19.4
		CV	0.04	0.04	0.13	0.11
		SE	132.96	56.94	0.04	1.11

**Table 2:** Significant relationships between sensor measurements and plant parameters of wheat and barley, indicated by coefficients of determination (R²) at * *p* ≤ 5%, ** *p* ≤ 1%. Relationships are indicated for different indices. The closest relationships are indicated in bold.

	GreenSeeker		Passive Spectrometer				
**Barley** ZS 32	774/656	NDVI	774/656	NDVI	800/770	820/755	720/400
Fresh weight	0.53 **	0.48 **	0.49 **	0.37*	0.86 **		
Dry weight	0.41 **	0.35 *	0.37 *	0.28 *	0.85 **		
N-content							
N-uptake	0.46 **	0.46 **	0.44 **	0.43 **	0.84 **		
**Barley** ZS 65							
Fresh weight	0.25 *	0.34 *	0.85 **	0.70 **			
Dry weight		0.20 *	0.74 **	0.61 **			
N-content		0.21 *					
N-uptake	0.21 *	0.29 *	0.71 **	0.50 **			
**Barley** ZS 85							
Fresh weight	0.50 **	0.45 **	0.67 **	0.64 **		0.77 **	
Dry weight	0.46 **	0.44 **	0.60 **	0.64 **		0.72 **	
N-content	0.20 *		0.30 *			0.27 *	
N-uptake	0.43 **	0.35 **	0.63 **	0.53 **		0.71 **	
**Wheat** ZS 32							
Fresh weight	0.62 **	0.60 **	0.86 **	0.52 **			
Dry weight	0.63 **	0.58 **	0.88 **	0.54 **			
N-content							
N-uptake	0.59 **	0.55 **	0.93 **	0.55 **			
**Wheat** ZS 65							
Fresh weight	0.74 **	0.69 **	0.67 **	0.60 **			
Dry weight	0.72 **	0.70 **	0.65 **	0.59 *			
N-content							
N-uptake	0.51 *	0.47 *	0.37 *	0.33 *			
**Wheat** ZS 85							
Fresh weight							0.66 **
Dry weight							0.63 **
N-content							
N-uptake							0.40 *
